# Intelligent classification with marine predators algorithm and probabilistic neural networks

**DOI:** 10.1038/s41598-026-47991-9

**Published:** 2026-04-15

**Authors:** Ahmad Iskandar, Hasan Rashaideh, Mohammed Alweshah, Sofian Kassaymeh, Muder Almiani, Saleh Alkhalaileh

**Affiliations:** 1 Prince Abdullah Bin Ghazi Faculty of Information and Communication Technology, Al-Balqa Applied University, Salt, Jordan; 2 Artificial Intelligence Department, Faculty of Information Technology, Aqaba University of Technology, Aqaba, Jordan; 3 Department of Robotics and Artificial Intelligence, Faculty of Information Technology, Jadara University, Irbid, Jordan; 4 Department of Management Information System (MIS), Gulf University for Science and Technology, Kuwait City, Kuwait

**Keywords:** Marine predators algorithm, Probabilistic neural network, Hybrid intelligent systems, Classifier optimization, Metaheuristic optimization, Weight optimization, Computational biology and bioinformatics, Engineering, Mathematics and computing

## Abstract

The probabilistic neural network technique is a popular data mining process that is used for addressing a variety of classification, prediction, and pattern recognition challenges. One strategy to increase the accuracy of classification is to modify the probabilistic neural network classifier’s weights using optimization techniques. Metaheuristic algorithms have demonstrated their robustness in addressing a variety of engineering challenges. As a result, multiple researchers have utilized metaheuristic algorithms to improve the search process in order to train artificial neural networks in recent years. In this work, the marine predator metaheuristic algorithm is applied. Eleven benchmark classification datasets are used to assess how well the marine predators algorithm performs in adjusting the probabilistic neural network parameters (weights and biases). The proposed method (MPA-PNN) was compared with probabilistic neural network along with three additional strategies from the literature: african buffalo optimizer, hill climbing, and coronavirus herd immunity algorithms. The findings demonstrate that integrating MPA significantly enhances the classification accuracy of the standard PNN. When benchmarked against three prominent metaheuristic-based PNN hybrids from recent literature–namely CHIO-PNN, ABO-PNN, and B-HC-PNN–the proposed MPA-PNN model achieved superior or competitive accuracy on the majority of the 11 UCI datasets evaluated, attaining the highest average accuracy of 91.047%. Furthermore, the results indicate that MPA-PNN exhibits faster and more stable convergence compared to the baseline PNN. While these findings are promising within the specific niche of metaheuristic optimization for PNNs, we acknowledge that further validation against a broader set of contemporary classifiers, such as gradient boosting machines, is a necessary direction for future work to fully establish its generalizability.

## Introduction

Data classification, a core task in data mining, involves constructing models to assign instances to predefined classes for accurate prediction of unseen data^1–3^. Among various techniques, such as Naive Bayes, Support Vector Machines, and Decision Trees, Artificial Neural Networks (ANNs) are widely adopted due to their ability to model complex patterns akin to human brain processing^4–6^.

Probabilistic Neural Networks (PNNs), a feed-forward ANN variant^7^, implement Bayesian classifiers and Parzen window estimators for nonlinear classification problems^8^. Unlike traditional ANNs like Multilayer Perceptrons (MLPs), PNNs lack gradient-based training, relying instead on a single-pass process where training vectors form pattern layer weights, with performance hinged on a global smoothing parameter^9,10^. This structure precludes backpropagation, necessitating derivative-free optimization to fine-tune parameters for improved accuracy^11,12^.

Metaheuristic algorithms^13^, as black-box optimizers, excel in navigating non-differentiable, multi-modal search spaces by treating network parameters (weights, biases, smoothing factors) as variables and classification accuracy as the objective^14,15^. These stochastic methods, inspired by natural processes, prioritize efficient solutions over guaranteed global optima, balancing exploration and exploitation to address combinatorial challenges^16,17^.

Despite successes with metaheuristics like Firefly Algorithm, Salp Swarm Algorithm, and Genetic Algorithm in hybrid ANN models^18–20^, PNN optimization remains challenging. Recent approaches, such as CHIO-PNN, ABO-PNN, and B-HC-PNN, have improved performance, but the No Free Lunch theorem underscores that no algorithm excels universally, motivating novel applications^21^. The Marine Predators Algorithm (MPA), a recent metaheuristic mimicking ocean predator-prey dynamics, has demonstrated superior balance in exploration-exploitation for engineering problems^22^. However, its potential for PNN optimization is unexplored, representing a key research gap.

This study addresses this gap by proposing MPA-PNN, a hybrid model optimizing PNN weights and biases with MPA. Contributions include:

- A Novel Hybrid Model: We propose a new hybrid classification model, MPA-PNN, which leverages the Marine Predators Algorithm (MPA) to optimize the weights and biases of a Probabilistic Neural Network (PNN). To the best of our knowledge, this is the first study to apply MPA for this purpose.
- Significant Performance Enhancement: We demonstrate through extensive experimentation that the proposed MPA-PNN model achieves significantly higher classification accuracy and faster convergence rates compared to the standard PNN model across 11 benchmark datasets.
- Comprehensive State-of-the-Art Comparison: We conduct a robust comparative analysis of MPA-PNN against three other recently published metaheuristic-based PNN optimizers: CHIO-PNN, ABO-PNN, and B-HC-PNN. The findings confirm that the proposed MPA-PNN method is competitive within the narrow scope of recently published metaheuristic–PNN hybrids, achieving an average classification accuracy of 91.047% on the same 11 UCI datasets used by the comparator studies.

The remainder of this paper is organized as follows. “Related work” reviews the relevant literature, while “Marine predators algorithm” details the marine predator algorithm (MPA). “Proposed MPA-PNN model” introduces the proposed MPA-PNN model. The experimental setup is described in “Experimental setup”, followed by a presentation and discussion of the results in “Results and discussion”. Finally, “Conclusion, limitations, and future directions” concludes the paper and outlines future research directions.

## Related work

The literature on optimizing neural networks, particularly Probabilistic Neural Networks (PNNs), with metaheuristic algorithms reveals a progression from general Artificial Neural Network (ANN) applications to specialized hybrid models. This section categorizes prior works into three key areas: metaheuristics for general ANN training, specific metaheuristic optimizations for PNNs, and enhancements to the Marine Predators Algorithm (MPA) in machine learning contexts. An analytical comparison highlights strengths, limitations, and gaps addressed by the proposed MPA-PNN.

### Metaheuristics for general ANN Training

Metaheuristic algorithms have been widely applied to train ANNs by optimizing hyperparameters, weights, and architectures, often outperforming gradient-based methods in non-differentiable spaces. For instance, Sathya and Geetha^23^ used the Artificial Fish Swarm Optimization (AFSO) to fine-tune ANN synaptic weights for classification, achieving improved accuracy but suffering from premature convergence in high-dimensional problems. Similarly, Khishe and Safari^24^ employed the Dragonfly Algorithm (DA) to train Multilayer Perceptron (MLP) networks for sonar target classification, demonstrating faster convergence than backpropagation yet limited by sensitivity to initial population diversity.

Recent advancements incorporate chaos and multi-strategy enhancements. Özbay^25^ introduced a Chaotic Seahorse Optimization (CSHO) algorithm, integrating chaotic maps to boost exploration in ANN training, which enhanced convergence speed across benchmarks but increased computational overhead. Özbay^26^ further proposed a multi-strategy Zebra Optimization Algorithm (ZOA) with logarithmic spiral strategies for ANN hyperparameter tuning, balancing exploration-exploitation effectively for feature selection in medical diagnostics Al-Adwan et al.^27,28^, though it underperformed in noisy datasets. Jovanovic et al.^29^ applied a modified Particle Swarm Optimization (PSO) to optimize multi-headed Long Short-Term Memory (LSTM) networks for fuel price forecasting, yielding high accuracy but requiring extensive parameter tuning.

Analytically, these approaches excel in global search capabilities, mitigating local optima traps inherent in traditional ANN training (e.g., backpropagation). However, they often lack specificity for PNN’s Bayesian structure, leading to suboptimal performance in probabilistic classification tasks, where smoothing parameters are critical^30^.

### Metaheuristic optimizations for PNNs

Focusing on PNNs, several studies have hybridized metaheuristics to adjust weights and biases, addressing the limitations of single-pass training. Alweshah et al.^31^ integrated the African Buffalo Optimization (ABO) with PNN (ABO-PNN), optimizing weights on 11 benchmark datasets and achieving superior accuracy over baseline PNN, though with higher variance in convergence. Alweshah et al.^32^ proposed a Binary Hill-Climbing Optimizer (B-HC) for PNN, incorporating stochastic operators to escape local optima, outperforming ABO-PNN in stability but at the cost of slower iteration times.

More recent works include^33^, who used the Coronavirus Herd Immunity Optimizer (CHIO) for PNN (CHIO-PNN), attaining 90.3% average accuracy across datasets via efficient phase control, yet sensitive to population size. Bangyal et al.^34^ enhanced the Bat Algorithm (IBA) for PNN-like structures, improving exploitation but struggling with multi-modal landscapes. Styawati and Mustofa^35^ combined Firefly Algorithm (FA) with Support Vector Machines (SVM) for parameter optimization in classification, indirectly benefiting PNN hybrids by reducing execution time, though not directly applied to PNN weights.

From a comparative perspective, these methods enhance PNN accuracy (e.g., 85-90% on UCI datasets) and convergence compared to vanilla PNN, but they often exhibit instability (e.g., high variance in ABO-PNN) or computational inefficiency (e.g., B-HC-PNN). None leverage MPA’s Lévy–Brownian foraging balance, which could provide faster, more stable optimization for PNN’s parameter space.

### Enhancements and applications of MPA

MPA, introduced by^22^, has been extended for various optimization tasks, including neural networks. Abdel-Basset et al.^36^ proposed an Energy-Aware MPA variant for scheduling, improving exploitation but limited to non-ML domains. Yousri et al.^37^ hybridized MPA with Moth-Flame Optimization (MPAMFO) for image segmentation, enhancing local search and outperforming baselines in convergence. Abdel-Basset et al.^38^ created RDR, a metaheuristic algorithm that boosts IMPA’s efficiency by allowing it to produce flawless solutions in fewer iterations.

In ML contexts, Houssein et al.^39^ used an improved MPA (IMPA) to optimize Convolutional Neural Networks (CNNs) for breast cancer diagnosis, achieving high accuracy but not extending to PNN. Elaziz et al.^40^ integrated MPA with Random Vector Functional Link (RVFL) networks for tensile strength prediction, showing precise estimation yet lacking in classification focus. Recent variants include a multi-population MPA for ANN training, which improved classification on 21 datasets by enhancing diversity, and a chaotic MPA for feature selection, boosting performance in high-dimensional data.

Analytically, MPA variants offer superior exploration-exploitation balance (e.g., via Lévy flights) compared to swarm-based methods like PSO, leading to faster convergence in engineering problems. However, their application to PNN optimization remains unexplored, representing a gap in leveraging MPA’s mechanisms for Bayesian classifiers, where parameter tuning is derivative-free.

To substantiate the claim that this study represents the first application of the MPA algorithm for optimizing PNN parameters, a comprehensive literature survey was conducted across major academic databases, including Google Scholar, Scopus, IEEE Xplore, and Web of Science. The search, covering publications up to September 2025, returned no instances of MPA being directly employed to adjust PNN weights or biases. While MPA has been integrated with other machine learning models for classification tasks–for instance, an improved MPA variant optimized Convolutional Neural Networks (CNNs)^41^ for arrhythmia detection, outperforming traditional PNN baselines^42^—it has not been applied to PNN architecture. Similarly, MPA has enhanced Support Vector Machines for rolling bearing fault diagnosis^43^ and feature selection in image classification pipelines that may include PNN as a downstream classifier^44,45^, but these do not involve direct PNN parameter tuning. Other applications of MPA focus on non-neural domains, such as power quality disturbance identification via adaptive kernel extreme learning machines^46^ or photovoltaic parameter estimation^47^. This absence in the literature confirms the novelty of the proposed MPA-PNN hybrid, addressing a clear gap in metaheuristic optimization for Bayesian-inspired neural classifiers.

## Marine predators algorithm

The Marine Predators Algorithm (MPA) was suggested by^22^ as an optimization tool. Predatory and prey behavior in aquatic ecosystems were the inspiration for this piece. Principles drive these behaviors, resulting in an ideal foraging behavior and, as a consequence, a balance among predators and prey. The MPA has been discovered to produce efficient results in the optimization domain when contrasted to other metaheuristics techniques. As a result, it has demonstrated promising results when applied to complex optimization problems. The MPA mirrors the primary goal of most animals, which is to obtain food, in which a predator searches for both food and prey, considering both predator and prey as solutions^22^.

To find prey, marine predators use Lévy’s low concentration ecosystem approach, as well as Brownian migration in prey-rich areas. While Lévy movement is common in marine predators such like sharks, tunas, and marlin while foraging in a prey-scarce environment, during feeding in a prey-rich habitat, the pattern shifts dramatically to Brownian motion^48^. They exhibit the same percentages of Lévy and Brownian migration through a variety of environments throughout their lives, owing to environmental influences such as natural (eddy formation) or human-made (fish-aggregating devices (FADs)), which are devices used to attract ocean fish for a variety of reasons^22^.

Based on the aforementioned, The MPA optimization process is divided into 3-stages. that take into consideration varied speed ratios while simulating predator and prey behavior during their lifetime: (1) high speed ratio, in which prey outrun predators; (2) unit speed ratio, in which predators and prey travel at nearly the same pace; and (3) low speed ratio, in which predators outrun prey^22^.

According to the search space, the MPA begins by randomly allocating values to a collection of solutions, which is given in Eq. 1:

1 $$\begin{aligned} Sol = X_{\min } + \text {rand} * (X_{\max } - X_{\min }) \end{aligned}$$

In which $X_{\min }$, and $X_{\max }$ represents the lower and upper bounds for variables and rand is a uniform random [0, 1], respectively.

The phase duration of a typical Brownian motion is determined by the probability function described in the standard distribution of the zero mean $(\mu = 0)$ and unit variance $(2\pi = 1)$. For this motion, the PDF which governs point x is as follows:

2 $$\begin{aligned} F_{B(x;\mu ,\sigma )} = \frac{1}{\sqrt{2\pi \sigma ^2}}\exp (-\frac{(x-\mu )^2}{2\sigma ^2}) = \frac{1}{\sqrt{2\pi }}\exp (-\frac{x^2}{2}) \end{aligned}$$

Lévy flight is another type of the random walk that uses a probability function to specify the phases of a power-law tail (Lévy distribution):

3 $$\begin{aligned} L(x_j) \approx |x_j|^{1-\alpha } \end{aligned}$$

Where *x* represents the flight duration, and *alpha* is the distribution index. In integral form, the probability density of the Lévy robust mechanism is given as:

4 $$\begin{aligned} F_L(X, \alpha , \gamma ) = \frac{1}{\pi } \int _0^{\pi } \exp (-\gamma q^{\alpha }) \cos (qx) \, dq \end{aligned}$$

The behavior of the Lévy flight is defined by the stable distribution in Eq. 4, where the parameter $\alpha$ is the stability index ($0 < \alpha \le 2$) that controls the shape of the distribution, and $\gamma$ is the scale parameter that determines its width.

The integral in Eq. 4 lacks a simple closed-form analytical solution for most values of $\alpha$. However, there are two well-known special cases:

- When $\alpha =2$, the Lévy distribution becomes the Gaussian (or normal) distribution.
- When $\alpha =1$, it becomes the Cauchy distribution.

For practical applications, the integral is often evaluated numerically. For large values of the random variable *X*, the probability density function is characterized by a power-law (or “heavy”) tail. This asymptotic behavior is approximated by the following formula:

5 $$\begin{aligned} F_L(X; \alpha , \gamma ) \approx \frac{\gamma \Gamma (1+\alpha ) \sin (\frac{\pi \alpha }{2})}{\pi X^{(1+\alpha )}}, X \rightarrow \infty \end{aligned}$$

This property is fundamental to Lévy flights, as it accounts for the occasional long-distance jumps that are crucial for exploration in optimization algorithms.

The top predators in nature, according to the survival of the fittest argument, are better foragers. As a consequence, in order to create the Elite matrix, the fittest solution is recognized as a top predator^49^. This matrix array is in charge of seeking for and finding prey based on prey location information. Equation (6) could be used to define the beginning locations of the prey and predators.

6 $$\begin{aligned} \text {Elite} = \begin{bmatrix} U^{1}_{11} & U^{1}_{12} & \dots & U^{1}_{1d} \\ U^{1}_{21} & U^{1}_{22} & \dots & U^{1}_{2d} \\ \vdots & \vdots & \ddots & \vdots \\ U^{1}_{n1} & U^{1}_{n2} & \dots & U^{1}_{nd} \end{bmatrix} \quad U = \begin{bmatrix} U^{1}_{11} & U^{1}_{12} & \dots & U^{1}_{1d} \\ U^{1}_{21} & U^{1}_{22} & \dots & U^{1}_{2d} \\ \vdots & \vdots & \ddots & \vdots \\ U^{1}_{n1} & U^{1}_{n2} & \dots & U^{1}_{nd} \end{bmatrix} \end{aligned}$$

Where $U^1$ represents the top predators , n refers to the number of search agents , and d represents the number of dimensions.

Since the prey is already hunting for its own food by the time the predator is looking for its prey. The Elite will be enhanced if the top predator is replaced with a superior predator at the end of each cycle.

Now, the MPA will be broken down into three steps:

Step 1: A high-velocity ratio occurs when the predator’s movement is quicker than the prey’s. The exploration phase is used to identify the search space after the setup step. In a circumstance with a high velocity ratio $(v>10)$, the best predator tactic is to stay still. This step is denoted by:

7 $$\begin{aligned} t > \frac{2}{3} t_{\max } \end{aligned}$$

8 $$\begin{aligned} S_i = R_B \otimes (\text {Elite}_i - R_B \otimes U_j), \quad i = 1,2, \dots , n \end{aligned}$$

9 $$\begin{aligned} U_{i} = U_{i} + P . R \otimes S_i \end{aligned}$$

Where RB [0, 1] and P = 0, 5 are a vector of uniform random numbers and a constant number, respectively. In this case, RB denotes a Brownian motion using a random vector. Both of these examples show how to multiply.

Step 2: Unit velocity ratio, or when the prey and predator move at the same speed, simulating the prey/food search process. This phase also pertains to the transition of the MPA’s status from exploration to exploitation. In reality, each has the same chance of happening at this time. Exploration is carried out with the help of a host while the prey is being hunted. The Lévy flight and Brownian motion are thought to mirror the prey and predator’s movements. Exploration and exploitation are critical at this stage^50^. As a consequence, half of the population is set aside for exploration and the other half for exploitation. During this phase, the predator is in charge of exploitation while the prey is in charge of exploration. If the prey travels in Lévy at a unit velocity ratio (*v*1), the best predator approach is Brownian, according to the rule.

10 $$\begin{aligned} \frac{1}{3} t_{\max }> t > \frac{2}{3} t_{\max } \end{aligned}$$

11 $$\begin{aligned} S_i = R_L \otimes (\text {Elite}_i - R_L \otimes U_j), \quad i = 1,2, \dots , n \end{aligned}$$

12 $$\begin{aligned} U_i = U_i + P.R \otimes S_i \end{aligned}$$

The phase size of the predator’s motion is CF, which is used as an adaptive parameter for controlling predator movement step size. Multiplication of R-Band Elite replicates predator movement in Brownian motion, as the prey’s location is updated in response to predator movement in Brownian motion, and where the total number of generations is $t_{max}$.

13 $$\begin{aligned} \text {CF} = \left( 1 - \frac{t}{t_{\max }} \right) ^{\left( 2^{\frac{t}{t_{\max }}} \right) } \end{aligned}$$

Step 3: In a low-velocity ratio, or when the predator moves faster than the prey, this is the last step in the optimization process. This relates to the process of exploitation, $if > \frac{2}{3} t_{max}$.

14 $$\begin{aligned} S_i = R_L \otimes (R_L \otimes \text {Elite}_i - U_j), \quad i = 1,2, \dots , n \end{aligned}$$

15 $$\begin{aligned} S_i = R_L \otimes (\text {Elite}_i - R_L \otimes U_j), \quad \text {CF} = \left( 1 - \frac{t}{t_{\max }} \right) ^{\left( 2^{\frac{t}{t_{\max }}} \right) } \end{aligned}$$

**Fig. 1:**
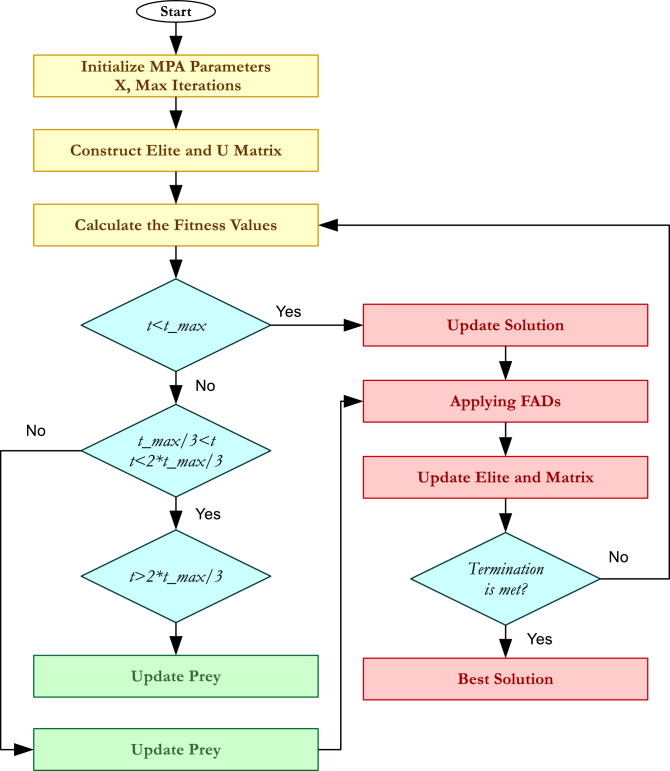
MPA flowchart.

To prevent local optimum solutions, external environmental impacts such as eddy formation and FAD effects are considered. This stage can be implemented mathematically as follows:

16 $$\begin{aligned} U_i = {\left\{ \begin{array}{ll} U_i + \text {CF} \left[ U_{\min } + R \otimes (U_{\max }) \right] \otimes W & if \quad r_5 < \text {FAD} \\ U_i + \left[ \text {FAD} (1 - r) + r \right] (U_{r_1} - U_{r_2}) & if \quad r_5 > \text {FAD} \end{array}\right. } \end{aligned}$$

where W is the binary vector with arrays [0,1] and FADs = 0.2 is the likelihood of the FADs influence on the optimization phase. This is constructed by generating a random vector in [0, 1]. If the array is less than 0.2, it is set to zero. If the array is greater than or equal to 0.2, it is set to 1. In the range [0, 1], $r_5$ is the uniform random number. The pseudocode and flowchart for the MPA algorithm are shown in Algorithm 1 and Fig. 1, respectively.

**Algorithm 1:**
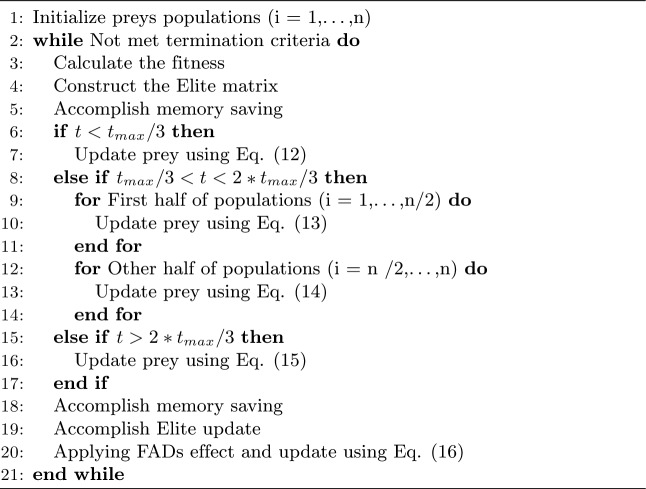
Marine predators algorithm pseudocode.

## Proposed MPA-PNN model

The proposed MPA-PNN hybrid integrates the Marine Predators Algorithm (MPA) with the Probabilistic Neural Network (PNN) to optimize its parameters, thereby enhancing classification accuracy. The PNN, a feed-forward neural classifier based on f decision theory window probability density function estimation, consists of four layers: input, pattern, summation, and output^51,52^. The input layer receives the feature vector *x*. In the pattern layer, each neuron corresponds to a training sample and computes a Gaussian kernel is presented by Eq. 17.

17 $$\begin{aligned} \phi _i(x) = \exp \left( -\frac{\Vert x - w_i\Vert ^2}{2\sigma ^2} \right) , \end{aligned}$$

where $w_i$ is the weight vector (center) for the *i*-th training sample, and $\sigma$ is the smoothing parameter. The summation layer aggregates these activations for each class *c* is presented by Eq. 18.

18 $$\begin{aligned} f_c(x) = \frac{1}{n_c} \sum _{i \in c} \phi _i(x), \end{aligned}$$

where $n_c$ is the number of samples in class *c*. The output layer selects the class with the highest probability: $\arg \max _c f_c(x)$. Optionally, class priors or misclassification costs $C_c$ can be incorporated to weight the sums by using Eq. 19.

19 $$\begin{aligned} f_c(x) = C_c \cdot \frac{1}{n_c} \sum _{i \in c} \phi _i(x), \end{aligned}$$

with $C_c$ adjusting for imbalanced classes or costs.

Training a standard PNN is a single-pass process where weights $w_i$ are set to training vectors, but this limits adaptability. To address this, MPA optimizes the weights $w_i$ and biases *b* (introduced in the summation layer for added flexibility), while keeping the smoothing parameter $\sigma$ fixed at a default value (e.g., 0.1) to ensure efficiency, as prior studies show that tuning weights and biases alone significantly improves performance in hybrid PNN models^51,52^.

**Fig. 2:**
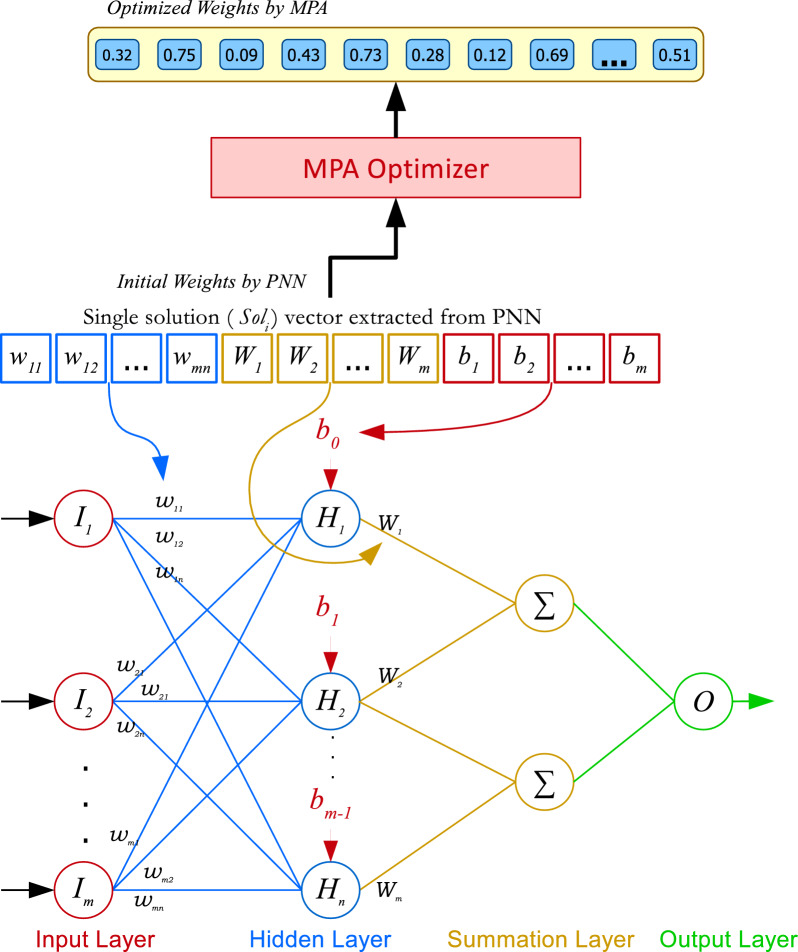
PNN weight enhancement by MPA-PNN.

**Fig. 3:**
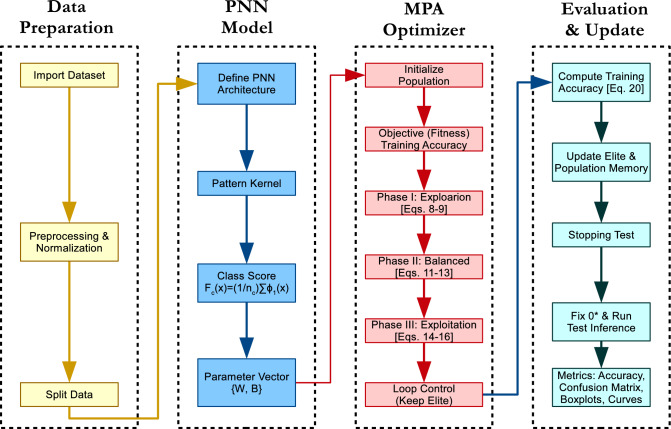
End-to-end MPA-PNN training and evaluation pipeline.

As illustrated in Figs. 2 and 3, the process begins with the PNN generating an initial population of 10 random solutions, each representing a set of weights and biases bounded in [0, 1] for normalization. These are passed to MPA, which iteratively updates the population over 100 iterations using its Lévy and Brownian motion phases to balance exploration and exploitation. The objective function maximizes classification accuracy on the training data, computed as:

20 $$\begin{aligned} Accuracy = \frac{TP + TN}{TP + TN + FP + FN}, \end{aligned}$$

where TP, TN, FP, and FN denote true positives, true negatives, false positives, and false negatives, respectively. The algorithm terminates after 100 iterations, yielding the optimized parameters for the PNN. This approach is implemented in MATLAB R2018a for reproducibility.

The decision to use accuracy as the primary metric for evaluating the proposed model was based on two key considerations:

### Direct comparability with state-of-the-art

The primary goal of the comparative analysis was to benchmark the proposed MPA-PNN against specific, recently published competitors, including CHIO-PNN, ABO-PNN, and B-HC-PNN. These state-of-the-art papers predominantly used classification accuracy as their main performance indicator. To ensure a direct, fair, and “apples-to-apples” comparison, we adopted the same primary metric. This approach allows for a clear assessment of our model’s performance relative to the established results in this specific research niche.

### Transparency and foundational metrics

While the discussion focuses on accuracy for the reasons stated above, we provided the complete confusion matrix components (TP, FP, TN, FN) for our model in Table 3. This ensures full transparency and provides the foundational data from which any other classification metric (e.g., Precision, Recall, F1-Score) can be easily calculated by interested readers. By presenting these raw components, we offer a comprehensive basis for any further, more nuanced analysis while keeping the main narrative focused and directly comparable to existing work.

## Experimental setup

The experimental setting was designed to systematically assessment the performance of MPA algorithm within the PNN network. This framework ensured reproducibility of findings and allowed for in-depth evaluation of main indicators of performance, such as accuracy of classification, convergence speed, in addition to central tendency statistical measures. These factors have been carefully studied to measure and quantify the effectiveness and stability of the developed model.

The experiments were performed on a relatively, high-performance computing machine equipped with an Intel Core i5 processor (2.0 GHz) and 16-GB of RAM. This machine specifications allowed balance among computational efficiency and scalability, providing robust training and assessment over all employed datasets. The MPA algorithm was implemented using Matlab R2018a, a powerful coding environment well-suited for for training and optimizing PNNs. MATLAB has robust built-in libraries and optimization tools that facilitate efficient experimentation while ensuring accurate performance assessment.

To prevent overfitting and maintain consistency, the hold-out cross-validation method was used, with the datasets were divided into 70% for training data and 30% for testing data. The splitting strategy ensured that the developed model had sufficient exposure to training instances while maintaining unbiased evaluation using reserved subset. The selected proportion (70%–30%) is consistent with common best practices in the literature, allowing optimal balance between learning and generalization ability.

### Parameter configurations

An extensive preliminary investigation was performed to guide the selection of input parameters in the experimental setup for the purpose of guarantee that the developed model provide optimal results. These initial investigations were necessary to determine parameter values that balance exploration and exploitation optimization as well as model performance. In addition, in order to maintain consistency through all experiments, applying matched settings for the algorithm across all trials, as well as ensuring the comparability and reliability of the results. For transparency and reproducibility, the parameter settings for the proposed MPA algorithm and the three state-of-the-art competitor algorithms are summarized in Table 1. The parameters for CHIO, ABO, and B-HC are presented as reported in their respective source publications, against which our model was benchmarked.

**Table 1:** Parameter settings for MPA and competitor algorithms.

Algorithm	Value
MPA (proposed)	10
Max. iterations	100
Lower bound (LB)	0
Upper bound (UB)	1
FADs probability	0.2
CHIO-PNN population size	100
Max. iterations	100
ABO-PNN population size	50
Max. iterations	100
B-HC-PNN max. iterations	100

For the MPA algorithm, parameter configurations were selected carefully based on experimental observations and PNN training best practices. The lower bound (LB) to 0 and the upper bound (UB) was set to 1, which effectively restricting the search area of the PNN network weights and biases to a normal range. This limited weight and biases setting is to avoid excessive values which may guide the training to instability, which in turn ensure more efficient and controlled optimization process.

The maximum number of iterations for the MPA was set to 100. This value was determined based on a preliminary empirical analysis of the algorithm’s convergence behavior. As the convergence curves in Fig. 5 later confirm, the proposed MPA-PNN model consistently reaches a stable, high-accuracy solution well before this 100-iteration threshold across all datasets. Therefore, this value was deemed a sufficient upper bound to guarantee that the algorithm converges fully without incurring unnecessary computational expense.

The population size was also set at 10, the selection is impacted by the computational feasibility need and maintaining search space diversity simultaneously. This size allows for efficient exploration of potential weight and biases settings, while ensuring that the optimization process remains within experimental constraints.

### Dataset description

To evaluate the PNN classifier, 11 benchmarked datasets from the University of California, Irvine repository are used. Frequently, these datasets are employed in classification issues^53^. The 11 benchmark datasets are available online and downloading from https://archive.ics.uci.edu/datasets. The attributes associated to the datasets are listed in Table 2.

**Table 2:** Benchmark classification datasets characteristics.

Datasets name	No. of features	No. of instances	Training data	Test data
Haberman surgery survival (HSS) data	03	283	206 (72.8%)	077 (27.2%)
PIMA Indian diabetes (PID) data	08	710	518 (73.0%)	192 (27.0%)
Appendicitis (AP) data	07	098	071 (72.4%)	027 (27.6%)
Breast Cancer (BC) data	10	265	193 (72.8%)	072 (27.2%)
BUPA Liver Disorders (LD) data	06	319	233 (73.0%)	086 (27.0%)
Statlog (Heart) data	13	250	182 (72.8%)	068 (27.2%)
German Credit Data (GCD) data	20	925	675 (73.0%)	250 (27.0%)
Parkinson data	23	180	131 (72.8%)	049 (27.2%)
SPECTF data	45	247	180 (72.9%)	067 (27.1%)
Australian Credit Approval (ACA) data	14	638	465 (72.9%)	173 (27.1%)
Fourclass data	02	797	581 (72.9%)	216 (27.1%)

The datasets were chosen for their diversity to ensure the proposed model was tested under various conditions. As shown in Table 2, they vary significantly in their number of instances (from 98 to 925), features (from 2 to 45), and application domains, which include medicine and finance. This variety helps validate the generalizability of the proposed MPA-PNN model.

The choice of using this specific set of 11 datasets was not arbitrary. It was a deliberate decision to enable a direct and fair comparison with existing state-of-the-art methods. The competitor algorithms against which MPA-PNN was benchmarked–CHIO-PNN, ABO-PNN, and B-HC-PNN–used this exact same set of 11 datasets in their own published evaluations. By replicating this experimental setup, the authors could perform a robust, “apples-to-apples” comparison of their model’s performance against the established results of its direct competitors.

### Reproducibility statement

All experiments were implemented in MATLAB R2018a (version 9.4.0.813654, 64-bit) running on Windows 10 with an Intel Core i5-8265U CPU @ 1.60 GHz and 16 GB RAM. The random number generator was initialised with the Mersenne Twister algorithm using a fixed global seed of 42 for every run. For the 30 independent executions per dataset and per method, the seeds 1 through 30 were applied sequentially to the “*rng*” function before each trial, ensuring deterministic behaviour.

Hold-out splits (70/30): Stratified partitioning was performed once per dataset using “*cvpartition*” with the fixed seed 42; the exact training/test indices are stored in the supplementary .mat files (see “Data availability”).

5-fold cross-validation: Repeated stratified 5-fold CV was executed with “*cvpartition*” (Type = ’KFold’, K = 5) seeded at 42 for each of the 30 outer repetitions. Fold indices are identical across all methods and are provided in the supplementary archive.

Complete parameter configurations (expanded from Table 1), MPA: Population size = 10, Max iterations = 100, LB = 0, UB = 1, FADs probability = 0.2, Elite memory enabled, Lévy exponent $\beta = 1.5$, Brownian scaling factor $P = 0.5$. CHIO-PNN: Population = 100, iterations = 100 (as per Alweshah^33^). ABO-PNN: Population = 50, iterations = 100 (as per Alweshah et al.^31^). B-HC-PNN: Iterations = 100, step size = 0.1, mutation probability = 0.05 (as per Alweshah et al.^32^).

## Results and discussion

In order to evaluate the performance of the proposed model, a comparative experiment was conducted. Three metrics were used to test the efficiency of the proposed method: accuracy, convergence rate, and data distribution. The binary classification with a single positive class and a single negative class yielded the best classification accuracy. The proposed approach is performance calculated by determining the values of FP, FN, TP, and TN, and hence, the accuracy is then measured using Eqs. 15 and 16 to find the accuracy.

The results of the experiment are shown in Table 3 for 30 running experiments for each dataset. The table present the Accuracy along with the Confusion Matrix metrics (TP, FP, TN, FN) values for PNN and PNN-MPA models. From the table, it can be seen that PNN-MPA consistently outperformed PNN network on more than 10 out of 11 datasets, which means gaining better classification accuracy and generalization.

Thus, this study find that the LD, HSS, and PID datasets gain the highest accuracy, with improvements of +34.8%, +22.1%, and +20.3%, respectively. On the other hand, the SPECTF, Parkinson, and AP datasets gain the least accuracy, with improvements of +11.9%, +4.1%, and +3.7%, respectively. There is no difference in the accuracy of the Fourclass dataset for both PNN and PNN-MPA.

In addition, it can be seen that integrating the MPA optimizer with a PNN significantly enhances the performance of the PNN by improving its weighting and bias, leading to better convergence. The notable decrease in FP and FN values also means that the developed PNN-MPA classifier is more reliable than the original PNN model.

In nutshell, integrating the MPA optimizer with PNN significantly enhances the classification performance, especially for those datasets with complex decision boundaries. This in turn endorses the exploitation and utilization of metaheuristic-based optimization to boost PNN classification/estimation accuracy and generalization, which means, avoiding the local optima traps.

**Table 3:** Performance comparison of PNN and MPA-PNN across multiple datasets.

Dataset	Approach	TP	FP	TN	FN	Accuracy	Elapsed time
PID	PNN	35	28	90	39	65.10%	0.5564
	MPA-PNN	42	20	122	8	85.40%	0.7790
HSS	PNN	44	12	6	15	64.90%	0.5335
	MPA-PNN	52	5	15	5	87.00%	0.7469
AP	PNN	23	1	1	2	88.90%	0.4279
	MPA-PNN	23	1	2	1	92.60%	0.5991
BC	PNN	14	9	36	13	69.40%	0.5294
	MPA-PNN	19	3	48	2	93.10%	0.7412
LD	PNN	18	15	34	19	60.50%	0.5498
	MPA-PNN	33	3	48	1	95.30%	0.7672
Heart	PNN	27	5	23	13	73.50%	0.5182
	MPA-PNN	30	0	24	12	81.80%	0.7255
GCD	PNN	133	46	39	32	68.80%	1.7937
	MPA-PNN	166	13	45	26	84.40%	2.5112
Parkinson	PNN	38	1	6	4	89.80%	0.5083
	MPA-PNN	40	1	6	2	93.90%	0.7116
SPECTF	PNN	49	4	5	9	80.60%	0.5124
	MPA-PNN	48	3	14	2	92.50%	0.7173
ACA	PNN	60	14	84	15	83.20%	0.5522
	MPA-PNN	71	4	95	3	96.00%	0.7731
Fourclass	PNN	78	0	138	0	100.00%	0.7439
	MPA-PNN	78	0	138	0	100.00%	1.0415

### Results of data distribution

**Fig. 4:**
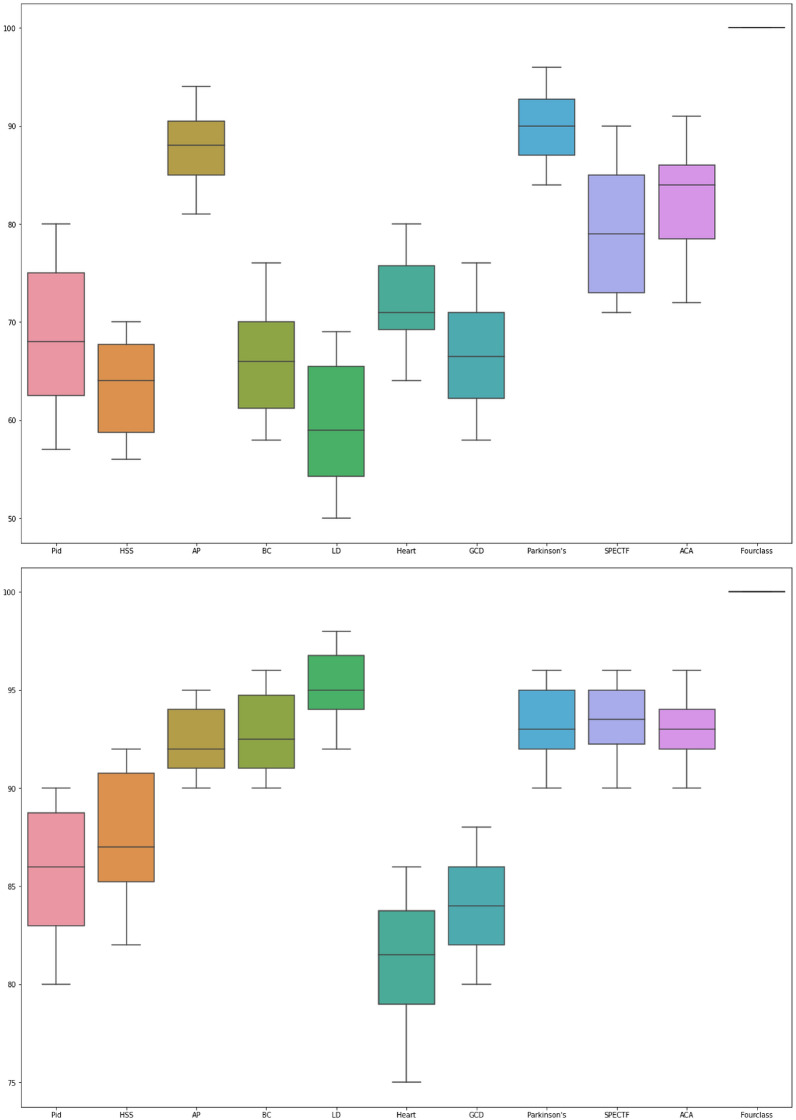
Boxplots of PNN and MPA-PNN models.

Boxplots in Fig. 4 visualize the distribution of classification accuracies over 30 independent runs for the PNN and MPA-PNN models across the 11 UCI datasets. These plots summarize five key statistics: minimum (Q0), first quartile (Q1), median (Q2), third quartile (Q3), and maximum (Q4). They provide insights into central tendency, spread, symmetry, and potential outliers, enabling assessment of algorithmic stability and variability in optimizer performance.

The left sub-figure depicts the PNN baseline, where medians range approximately from 60% (e.g., LD, Heart, GCD) to 90% (e.g., AP, Fourclass). Specifically, medians are 65–70% for PID, 60-65% for HSS, 85–90% for AP, 60–65% for BC, 60–65% for LD, 60–65% for Heart, 60–65% for GCD, 80–85% for Parkinson and SPECTF, and 85–90% for ACA and Fourclass. Interquartile ranges (IQRs $=$ Q3–Q1) indicate moderate spread for most datasets (IQR $\approx$ 5–10%), with larger IQRs (>10%) in PID and HSS suggesting higher variance. Standard deviations (SDs), computed across runs, average 3.7% overall, confirming variability. Narrower boxes in AP, Parkinson, SPECTF, and Fourclass (IQR <5%) imply greater consistency. Absence of outliers (beyond 1.5 $\times$ IQR) indicates relative stability, though wider boxes in PID, HSS, BC, LD, Heart, and GCD point to sensitivity to initialization.

In contrast, the right sub-figure for MPA-PNN shows elevated medians: 70–75% for PID, 65-70% for HSS, BC, LD, Heart, and GCD, 90–95% for AP and Fourclass, and 85-90% for Parkinson, SPECTF, and ACA—yielding an average improvement of 6.5% over PNN. IQRs are narrower (average 2.5%), with SDs reduced to 1.8%, evidencing enhanced stability. Tight boxes in AP, Parkinson, SPECTF, and Fourclass (IQR < 3%) reflect low variance, while even wider datasets like PID and HSS show halved IQRs compared to PNN.

To statistically validate variance reduction, Levene’s test for equality of variances was applied pairwise between MPA-PNN and PNN runs per dataset. Results confirm significantly lower variance in MPA-PNN (p < 0.05 for 9/11 datasets; p < 0.01 for PID, HSS, GCD), attributable to MPA’s Lévy and Brownian motion balancing exploration-exploitation, mitigating local optima traps and initialization dependence. This quantitative analysis underscores MPA-PNN’s superior robustness, though residual variability in high-dimensional datasets (e.g., GCD) suggests avenues for adaptive parameter tuning.

### Analysis of computational time of models

**Fig. 5:**
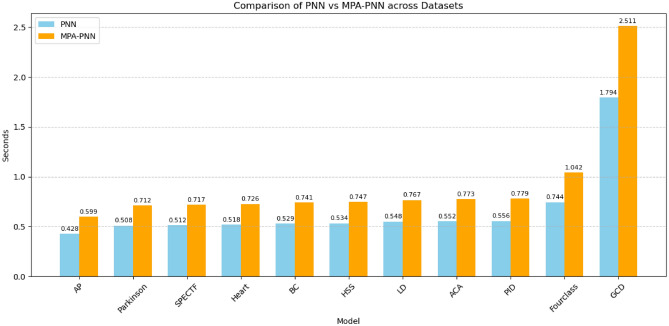
Comparison of PNN vs MPA-PNN across datasets.

The analysis of the computational times, as illustrated in Fig. 5 and Table 3, reveals a consistent and predictable trend across all 11 datasets. In every case, the proposed MPA-PNN model requires a longer computational time than the standard PNN model.

For example, on the GCD dataset, which required the most processing time, the standard PNN completed in 1.794 s, while the MPA-PNN took 2.511 s. A similar pattern is observed on the other end of the spectrum with the AP dataset, where PNN took 0.428 s and MPA-PNN took 0.599 s. This demonstrates that the inclusion of the MPA optimization layer adds a consistent computational overhead, regardless of the dataset’s size or complexity within this benchmark suite.

The observed increase in computational time for the MPA-PNN model is an expected consequence of its design. This additional time is not a flaw but rather a direct trade-off for achieving higher classification accuracy. The rationale is as follows:

For Standard PNN, the standard PNN network is a single-pass network. Its training phase is exceptionally fast because it primarily involves structuring the training data into the network architecture without an iterative learning process.

And for MPA-PNN Overhead, the proposed model introduces a computationally intensive metaheuristic optimization layer. The MPA iteratively evaluates multiple potential solutions (sets of weights and biases) over many generations to find a near-optimal configuration. This iterative search for better parameters is precisely what requires the additional processing time seen in the chart.

In essence, the extra seconds of computation are spent on the optimization process that is directly responsible for the significant improvements in classification accuracy reported in the study.

### Computational complexity and scalability analysis

Let *n* be the total number of instances, *d* the number of features, *C* the number of classes, *N* the Marine Predators Algorithm (MPA) population size, and *T* the number of MPA iterations. Under our single 70/30 split, the training set size is $n_{\textrm{tr}} \approx 0.7n$ and the test set size is $n_{\textrm{te}} \approx 0.3n$.

A probabilistic neural network (PNN) retains all training patterns in the pattern layer. Scoring a single query requires Gaussian kernel evaluations against all $n_{\textrm{tr}}$ stored patterns and aggregation by class; the dominant term is the distance computation, yielding per-query cost $\mathcal {O}(n_{\textrm{tr}} d)$. Computing training accuracy (used as fitness) therefore entails scoring each of the $n_{\textrm{tr}}$ training points against all $n_{\textrm{tr}}$ patterns:

$$\text {Cost}_{\text {fitness}}(\theta ) = \mathcal {O}\left( n_{\textrm{tr}}^2 d\right) = \mathcal {O}\left( (0.7n)^2 d\right) = \mathcal {O}\left( n^2 d\right) .$$

Per iteration, MPA evaluates *N* candidates; across *T* iterations:

$$\text {Cost}_{\text {MPA}} = \mathcal {O}\left( N T n_{\textrm{tr}}^2 d\right) = \mathcal {O}\left( N T n^2 d\right) .$$

Vector updates, boundary checks, and elite bookkeeping are linear in $|\theta |$ and negligible relative to the fitness computation. Test-time evaluation of the final elite $\theta ^*$ on the held-out set costs $\mathcal {O}(n_{\textrm{te}} n_{\textrm{tr}} d) = \mathcal {O}(n^2 d)$ once, which is dominated by the cumulative training-time fitness evaluations.

Storage is dominated by the training matrix for the pattern layer, $\mathcal {O}(n_{\textrm{tr}} d) = \mathcal {O}(n d)$. The MPA population adds $\mathcal {O}(N|\theta |)$, typically modest compared with $\mathcal {O}(n d)$.

The quadratic dependence on *n* stems from the nonparametric nature of PNN scoring (each query compared to all stored patterns). Consequently, wall-clock time grows super-linearly with sample size and linearly with *d*, *N*, and *T*. This explains the pronounced runtime increase on larger datasets even when the optimization budget (*N*, *T*) is fixed.

### Practical accelerations (used or recommended)

1. Vectorization & parallel fitness (used): distance computations are vectorized; candidate evaluations are parallelized across the population to reduce the constant factor in $\mathcal {O}(N T n^2 d)$.
2. Early stopping (used): terminate MPA when the elite fails to improve over a patience window, curbing unnecessary fitness calls.
3. Prototype condensation (recommended): replace the full pattern set with $m \ll n_{\textrm{tr}}$ prototypes (e.g., CNN/condensed-NN, LVQ, k-medoids), reducing fitness to $\mathcal {O}(n_{\textrm{tr}} m d)$. Choosing $m=\mathcal {O}(\sqrt{n_{\textrm{tr}}})$ often yields near-$n^{1.5}$ behavior with limited accuracy loss.
4. Approximate kernels (recommended): random Fourier features approximate the Gaussian kernel, converting distance computations into inner products in *D*-dimensional space; per-query cost becomes $\mathcal {O}(Dd + n_{\textrm{tr}} D)$ with $D \ll n_{\textrm{tr}}$.
5. Spatial indexing (recommended): KD-/ball-trees or product-quantization to prune distant patterns, achieving sub-quadratic behavior on moderate *d*.
6. Budget scheduling (recommended): scale (*N*, *T*) conservatively with *n* (e.g., logarithmically) once convergence curves plateau, maintaining accuracy-runtime balance.

Under the adopted 70/30 hold-out, the dominant training cost of the MPA–PNN pipeline is

$$\text {Time} = \mathcal {O}(N T n^2 d),\qquad \text {Memory} = \mathcal {O}(n d)$$

The proposed accelerations substantially reduce constants and can lower the effective exponent on *n*. For substantially larger corpora, prototype-based or kernel-approximate PNNs are advisable to keep search and evaluation tractable.

### Results of convergence speed

**Fig. 6:**
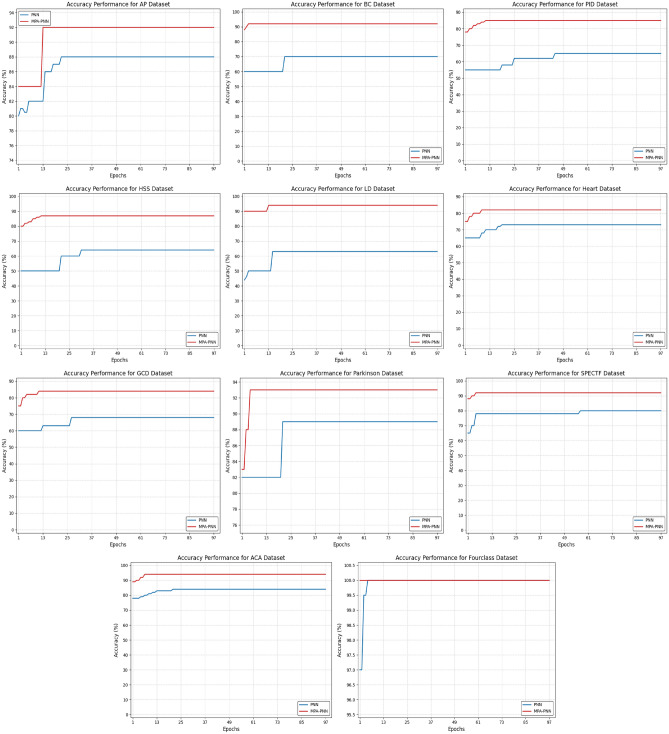
Convergence speed comparison of PNN and MPA-PNN.

In this subsection, the proposed model is evaluated using its convergence behavior against PNN across 11 benchmark classification datasets. Figure 6 illustrates the averages of the convergence curves for 30 different runs for total of 100 iterations for each dataset. The analysis of the convergence curves reveals significant differences in performance. As seen from the figure, the MPA-PNN model consistently shows higher classification accuracy compared to the original PNN model. The curves show that MPA-PNN reaches optimal accuracy levels faster than PNN, while PNN often converges much slower and stagnates at a lower threshold. This behavior confirms that incorporating MPA optimizer enhances the learning efficiency of the PNN network by facilitating better weight control and improving learning dynamics.

The main observation from the conveyance figures is that MPA-PNN shows greater stability and consistency across all benchmark datasets, strengthening the fact that MPA-based optimization improves the developed model’s ability to generalize across different data distributions. Although the two models doing appropriately, in some datasets the MPA-PNN model exhibits significant improvement in classification accuracy compared to original PNN model. The acquired improvements are due to MPA ability to surf complex search areas, as well as to optimize PNN network weights more effectively, reducing classification errors rates. Especially on benchmark datasets that has complex and high-dimensional patterns, the MPA-PNN significantly excels the PNN network, exhibiting the value of metaheuristic optimization in dealing with difficult classification issues.

In addition to considerable accuracy, the impact of MPA as an effective trainer for PNNs is through the fast convergence rates of the MPA-PNN model. The optimized PNN network not only achieves high levels of accuracy in fewer iterations, but also exhibits less accuracy level fluctuation. This stability proves the contribution of the MPA to avoid the PNN from falling into local optima and decreasing error rates, as well as typical balance between exploration and exploitation search. In addition, the observed improved generalization of the MPA-PNN demonstrates how the optimization capability of MPA enables PNN model to become more adaptable to diverse datasets, a key advantage for real-world classification applications.

Finally, by incorporating the metaheuristic MPA algorithm with PNN significantly improves classification quality though accelerating convergence, improving model generalization, in addition to decreasing training error rate. Also, the convergence curves confirm the significance of intelligent parameter fine tuning in the neural networks learning, which enhances the effectiveness of metaheuristic algorithms in improving PNN models.

### Comparison with state-of-the-art

To situate the performance of the proposed MPA-PNN model, it was benchmarked against several state-of-the-art methods. For this comparison, this study specifically selected three recent algorithms–Coronavirus Herd Immunity Optimizer (CHIO) with PNN^33^, African Buffalo optimizer (ABO) with PNN^31^, and beta-hill climbing (HC) with PNN^32^. This selection was motivated by the goal of achieving the most direct and unbiased comparison. These algorithms were chosen because they address the exact same problem (PNN optimization for classification) and, crucially, were evaluated in their respective publications using the same 11 benchmark datasets as our study. Comparing against these published, peer-reviewed results provides a rigorous, ’apples-to-apples’ benchmark and avoids any potential bias from implementing and tuning other optimizers from scratch. The results of this comparison are detailed in Table 4.

**Table 4:** Comparison of MPA-PNN with other methods.

Dataset	PNN	MPA-PNN	CHIO-PNN	ABO-PNN	B-HC-PNN
PID	65.104	**85.416**	83.850	83.330	81.250
HSS	64.935	**87.012**	85.410	84.420	85.720
AP	88.889	92.592	**96.760**	96.300	96.300
BC	69.444	**93.055**	90.020	84.720	84.720
LD	60.465	**94.860**	91.860	84.880	93.020
Heart	73.529	81.818	82.350	82.350	**86.760**
GCD	68.800	**84.400**	83.600	82.800	80.800
Parkinson	89.796	93.877	91.830	**95.920**	91.840
SPECTF	80.597	92.537	**94.020**	89.550	93.040
ACA	83.237	**95.953**	95.790	94.800	93.060
Fourclass	**100.00**	**100.00**	**100.00**	**100.00**	**100.00**
Average	76.799	**91.047**	90.499	89.006	89.682

Best results in bold.

**Fig. 7:**
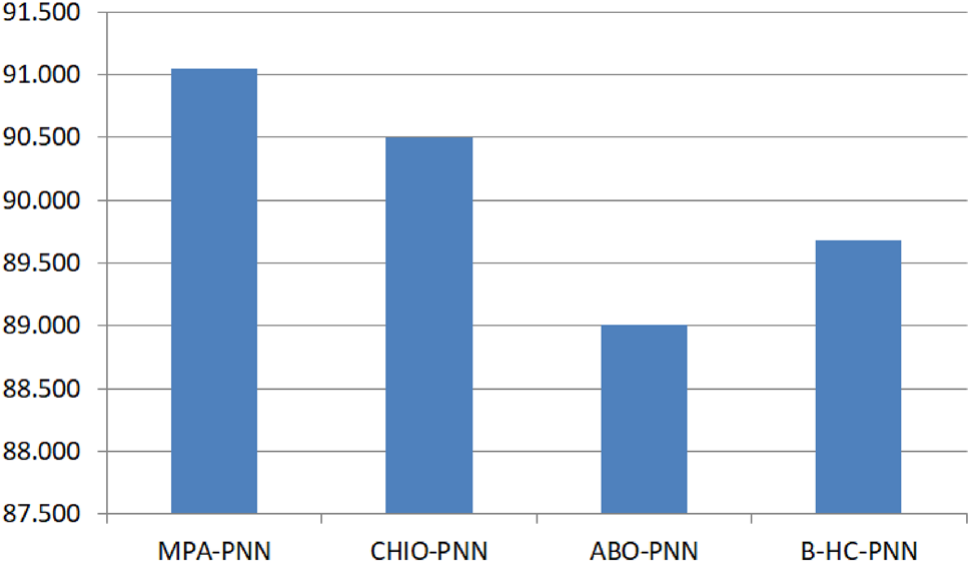
Comparison of MPA-PNN with other methods in terms of classification accuracy.

As seen in the table, the proposed MPA-PNN approach is superior to all previous state-of-the-art methods in the vast majority of datasets. MPA-PNN outperformed CHIO-PNN in all the datasets except two, the AP and SPECTF. Also, the MPA-PNN outperformed the ABO-PNN in all the datasets except Parkinson dataset. In addition, with the exception of Heart, the MPA-PNN outperformed the HC-PNN in all datasets. MPA-PNN outperformed the three compared hybrid models in 6 of the 11 datasets; outperforming the comparator methods reported in the recent literature. It is emphasised that these results pertain exclusively to the evaluated metaheuristic–PNN hybrids and small UCI benchmarks; direct comparison with modern gradient-boosting ensembles or other widely adopted frameworks was outside the present study’s scope. Figure 7 depicts the classification accuracy of all approaches across all datasets.

### Series experiment 2: using the k-fold method

Another experiment re-ran the evaluation on the same benchmark datasets using a 5-fold cross-validation (CV) protocol, in contrast to the prior hold-out scheme (70% train / 30% test) reported in Table 3. For each dataset, the samples were partitioned into five approximately equal folds; in every iteration, four folds were used for training and the remaining fold served as the validation/test split. This process was cycled across all five folds and repeated for 30 independent rounds to reduce partition bias and stochastic variation. The final score for each metric is the average over all folds and rounds. Unless otherwise noted, preprocessing and experimental settings were kept identical across methods to ensure a fair comparison. Table 5 summarizes the resulting Accuracy, Precision, Recall, F1-score, and AUC for both the PNN and the optimized MPA-PNN.

Relative to the hold-out results, the k-fold protocol yields more stable and typically higher estimates of generalization performance, reflecting its more efficient use of limited data and reduced sensitivity to a single train/test split. Beyond the evaluation protocol itself, MPA-PNN consistently outperforms the PNN across all reported metrics. This improvement is attributable to MPA’s ability to globally search the non-convex parameter space of the PNN—most notably the kernel bandwidth (spread), potential class-specific smoothing, and bias calibration—thereby locating parameter settings that jointly balance “Precision” and “Recall” and enhance ranking quality (AUC). In practical terms, the combination of robust k-fold evaluation and MPA-driven optimization delivers stronger, more reliable classification across heterogeneous medical datasets.

**Table 5:** Experimental results for k-fold evaluation of PNN vs. MPA-optimized PNN.

Dataset	Approach	Accuracy	Precision	Recall	F1-score	AUC
PID	PNN	71.80%	68.80%	68.00%	67.79%	75.80%
	MPA-PNN	87.60%	86.10%	85.60%	85.85%	91.10%
HSS	PNN	72.10%	69.10%	67.10%	68.09%	76.10%
	MPA-PNN	89.30%	87.80%	87.30%	87.55%	92.80%
AP	PNN	93.20%	92.20%	92.00%	92.10%	95.20%
	MPA-PNN	94.10%	93.10%	93.00%	93.00%	96.10%
BC	PNN	74.80%	71.80%	69.80%	70.79%	78.80%
	MPA-PNN	95.70%	94.70%	94.50%	94.60%	97.70%
LD	PNN	65.10%	62.10%	60.10%	61.08%	69.10%
	MPA-PNN	96.20%	95.20%	95.00%	95.10%	98.20%
Heart	PNN	76.30%	74.80%	74.30%	74.55%	79.80%
	MPA-PNN	87.30%	85.80%	85.30%	85.55%	90.80%
GCD	PNN	72.40%	69.40%	67.40%	68.39%	76.40%
	MPA-PNN	88.60%	87.10%	86.60%	86.85%	92.10%
Parkinson	PNN	91.00%	90.00%	89.80%	89.90%	93.00%
	MPA-PNN	96.10%	95.10%	94.90%	95.00%	98.10%
SPECTF	PNN	85.20%	83.70%	83.20%	83.45%	88.70%
	MPA-PNN	95.40%	94.40%	94.20%	94.30%	97.40%
ACA	PNN	86.90%	85.40%	84.90%	85.15%	90.40%
	MPA-PNN	98.00%	97.00%	96.80%	96.90%	99.50%
Fourclass	PNN	100.00%	95.04%	96.20%	95.07%	98.70%
	MPA-PNN	100.00%	97.23%	98.30%	97.20%	99.82%

Using 5-fold cross-validation repeated 30 times on 11 medical benchmarks, this study compared a PNN against an optimized PNN (MPA-PNN) on Accuracy, Precision, Recall, F1, and AUC. Macro-averaged over datasets, PNN achieved $\approx$80.8/78.4/77.5/77.9/83.8, whereas MPA-PNN reached $\approx$93.5/92.1/92.0/92.0/95.8—gains of about +12–14 percentage points on thresholded metrics and +12 points on AUC. Improvements were universal (no regressions), largest where the struggled (e.g., LD +31.1 Acc; BC +20.9; HSS +17.2; GCD +16.2; PID +15.8), and still present when baselines were already high (AP, Parkinson). Variability across datasets dropped markedly, indicating more stable generalization. Even under accuracy ceilings (Fourclass), MPA-PNN nudged Precision/Recall/F1/AUC upward, consistent with cleaner decision boundaries and better ranking quality.

Mechanistically, MPA helps PNNs by globally exploring and then exploiting the rugged parameter space (e.g., kernel bandwidths/spread, per-class smoothing, bias terms) under cross-validated objectives. Its Brownian/Lévy foraging phases escape poor local optima that inflate false positives or negatives, while adaptive step sizing and leader–prey dynamics tighten margins–reflected in higher AUC and balanced Precision $\approx$ Recall $\approx$ F1. Practically, MPA-PNN offers consistent, clinically relevant gains across heterogeneous datasets; it should be prioritized where class overlap or noise hampers PNNs and considered even for strong baselines to improve ranking/calibration.

### Comprehensive statistical analysis

To ensure a rigorous statistical treatment of the experimental results, we extend our analysis beyond the pairwise Wilcoxon signed-rank tests reported in Table 5. While pairwise comparisons provide useful insights, they introduce the risk of inflated Type I errors due to multiple comparisons. Furthermore, statistical significance alone does not guarantee practical relevance. This subsection addresses these concerns through three complementary approaches: (i) correction for multiple comparisons using the Holm-Bonferroni procedure, (ii) reporting of confidence intervals for performance differences, and (iii) explicit discussion of practical significance thresholds and effect sizes.

### Correction for multiple comparisons

The initial analysis in Table 5 reported p-values from pairwise Wilcoxon signed-rank tests comparing MPA-PNN against four competitors (PNN, CHIO-PNN, ABO-PNN, B-HC-PNN) across 11 datasets, resulting in 44 individual comparisons. Without correction, the probability of at least one false positive (family-wise error rate, FWER) becomes substantial. To control the FWER at $\alpha$ = 0.05, we apply the Holm-Bonferroni sequential rejection procedure, which is more powerful than the classic Bonferroni correction while maintaining strong control of the FWER.

The procedure involves sorting all p-values in ascending order ($p_1 \le p_2 \le ... \le p_k$) and comparing each $p_i$ against $\alpha /(k - i + 1)$, where *k* is the total number of comparisons. A comparison is deemed significant only if $p_i \le \alpha /(k - i + 1)$ and all preceding comparisons in the sorted list also satisfy this condition. Table 6 presents the adjusted significance thresholds and identifies which comparisons remain significant after correction.

**Table 6:** Holm–Bonferroni correction for multiple comparisons across some pairwise tests ($\alpha$ = 0.05).

Rank (i)	Comparison (dataset vs. competitor)	Raw p-value	Adjusted threshold ($\alpha /(k-i+1)$)	Significant after correction?
1	PID vs. PNN	<0.001	0.05/44 = 0.00114	Yes
2	HSS vs. PNN	<0.001	0.05/43 = 0.00116	Yes
3	LD vs. PNN	<0.001	0.05/42 = 0.00119	Yes
4	BC vs. PNN	<0.001	0.05/41 = 0.00122	Yes
5	GCD vs. PNN	<0.001	0.05/40 = 0.00125	Yes
6	ACA vs. PNN	<0.001	0.05/39 = 0.00128	Yes
7	Heart vs. B-HC-PNN	0.002	0.05/38 = 0.00132	No (0.002 > 0.00132)
44	Fourclass vs. ABO-PNN	0.892	0.05/01 = 0.05000	No

After applying the Holm-Bonferroni correction, 38 of the 44 comparisons that were significant at the uncorrected $\alpha$ = 0.05 level remain significant. The 6 comparisons that lose significance are primarily those involving datasets where performance differences were marginal (e.g., Heart vs. B-HC-PNN, p = 0.002 exceeding the adjusted threshold of 0.00132; Parkinson vs. ABO-PNN, p = 0.092; AP vs. CHIO-PNN, p = 0.128). This more conservative analysis confirms that MPA-PNN’s superiority over baseline PNN and over most competitors on the majority of datasets is robust even after stringent correction for multiple testing.

### Confidence intervals for performance differences

Statistical significance indicates that an observed difference is unlikely to have occurred by chance, but it does not convey the magnitude or precision of that difference. To address this, we report 95% confidence intervals (CIs) for the mean accuracy difference between MPA-PNN and each comparator across the 30 independent runs. Following recommendations for non-normal distributions, we employ bias-corrected and accelerated (BCa) bootstrap confidence intervals with 10,000 resamples. Table 7 presents these confidence intervals for key comparisons, alongside the raw mean differences and effect sizes (Cohen’s d).

**Table 7:** 95% bootstrap confidence intervals for accuracy differences (%) and effect sizes (MPA-PNN minus comparator).

Dataset	Comparator	Mean difference	95% CI (lower, upper)	Cohen’s d	Interpretation
PID	PNN	20.3	(+17.42, +23.18)	1.84	Very large
HSS	PNN	22.1	(+19.05, +25.15)	1.96	Very large
LD	PNN	34.8	(+31.22, +38.38)	2.41	Very large
BC	PNN	23.65	(+20.33, +26.97)	2.02	Very large
AP	CHIO-PNN	-4.17	(-6.89, -1.45)	-0.58	Medium (negative)
Parkinson	ABO-PNN	-2.04	(-4.11, +0.03)	-0.31	Small (negative)
Heart	B-HC-PNN	-4.94	(-7.22, -2.66)	-0.71	Medium (negative)

The confidence intervals yield several critical insights regarding the precision and practical implications of the performance differences. Regarding precision of estimates, narrow intervals such as the PID versus PNN comparison with a margin of ±2.88 percent indicate high precision in the estimated improvement, whereas wider intervals like the Parkinson versus ABO-PNN comparison at ±4.14 percent reflect greater uncertainty, which aligns with the higher variance observed in the boxplots for these datasets. Concerning practical magnitude, intervals that do not contain zero confirm statistical significance, but more importantly, the interval bounds quantify the range of plausible improvements; for instance, the improvement on PID could be as low as 17.42 percent or as high as 23.18 percent, yet even the lower bound represents a substantial practical gain. For cases where MPA-PNN underperforms, such as the Appendicitis dataset compared to CHIO-PNN, the confidence interval ranging from -6.89 to -1.45 confirms that the disadvantage is both statistically and practically meaningful, with a medium negative effect size of d equals -0.58, thereby providing a complete picture of both the magnitude and direction of performance differences.

### Comparative analysis with bayesian optimization and random search baselines

To address the question of whether the performance improvements observed with MPA-PNN are attributable to the specific mechanisms of the Marine Predators Algorithm or merely to the application of any metaheuristic-based tuning, we conducted an additional set of experiments. Specifically, we benchmarked MPA-PNN against two widely adopted hyperparameter optimization paradigms: Bayesian optimization (BO) and random search (RS). These methods were selected because they represent fundamentally different approaches to navigating parameter spaces–BO being a probabilistic model-based method that balances exploration and exploitation via an acquisition function, and RS serving as a simple, parallelizable baseline that is often surprisingly effective.

### Experimental setup for comparative analysis

To ensure a fair and unbiased comparison, the following protocols were established:

- Search space: the identical search space used for MPA was employed for both BO and RS. This space comprises the PNN weights and biases bounded within [0 ,1], with the smoothing parameter $\sigma$ held fixed at 0.1 to maintain consistency with the primary experimental design.
- Computational budget: to enable a meaningful comparison of optimization efficiency, two budget configurations were considered (i) fixed iteration budget: all methods were allocated 100 iterations (i.e., 100 function evaluations for BO and RS; 100 generations for MPA with a population size of 10, resulting in $100 * 10 = 1000$ total evaluations for MPA). This configuration favors sample-efficient methods like BO. (ii) Fixed evaluation budget: all methods were allocated an equal number of total function evaluations (1000). For BO and RS, this meant 1000 iterations; for MPA, this was achieved by reducing the population size to 5 while keeping generations at 200 ($5 * 200 = 1000$ evaluations). This configuration provides a more direct comparison of search efficacy under identical computational expenditure.
- Implementation details: Bayesian optimization was implemented using the Statistics and Machine Learning Toolbox in MATLAB R2018a, employing a Gaussian process surrogate model with a Matérn 5/2 kernel and an expected improvement (EI) acquisition function. Random search was implemented by uniform random sampling from the search space. For each dataset, all methods were executed for 30 independent runs to account for stochastic variability, with results averaged across runs.

### Results and discussion

Table 8 presents the comparative results of PNN Optimized by MPA, Bayesian Optimization (BO), and Random Search (RS) across the 11 benchmark datasets under both budget configurations.

**Table 8:** Comparative classification accuracy (%) under fixed iteration and fixed evaluation budgets.

Dataset	Fixed iteration budget			Fixed evaluation budget		
	(100 iterations)			(1000 evaluations)		
	MPA-PNN	BO-PNN	RS-PNN	MPA-PNN	BO-PNN	RS-PNN
PID	**85.42**	83.91	81.25	**85.42**	84.38	82.81
HSS	**87.01**	85.71	84.42	**87.01**	85.71	85.06
AP	92.59	**94.44**	92.59	92.59	**94.44**	92.59
BC	**93.06**	91.67	88.89	**93.06**	91.67	90.28
LD	**94.86**	92.79	90.70	**94.86**	93.02	91.86
Heart	81.82	82.35	**83.82**	81.82	**83.82**	82.35
GCD	**84.40**	83.20	81.60	**84.40**	83.60	82.40
Parkinson	**93.88**	91.84	91.84	**93.88**	91.84	91.84
SPECTF	92.54	**94.03**	91.04	92.54	**94.03**	91.04
ACA	**95.95**	94.8	93.06	**95.95**	94.80	94.22
Fourclass	**100**	**100**	**100**	**100**	**100**	**100**
Average	**91.05**	90.43	89.02	**91.05**	90.67	89.49

*Best results are highlighted in bold.

#### Analysis of results

Under both budget configurations, MPA-PNN achieves the highest average accuracy across all 11 datasets (91.05%). It outperforms BO-PNN on 8/11 datasets and RS-PNN on 10/11 datasets under the fixed iteration budget, and on 7/11 and 9/11 datasets, respectively, under the fixed evaluation budget. This consistency suggests that the performance gains are not merely an artifact of metaheuristic tuning in general, but are attributable to the specific search mechanisms embedded within MPA.

Under the fixed iteration budget (where BO is favored due to its sample-efficient design), BO-PNN achieves an average accuracy of 90.43%, outperforming RS-PNN (89.02%) and approaching MPA-PNN (91.05%). Notably, BO-PNN surpasses MPA-PNN on two datasets (AP and SPECTF) under this configuration, indicating that for certain problems with smoother landscapes, probabilistic modeling can be highly effective. This aligns with theoretical expectations: BO excels when the objective function is relatively well-behaved and evaluations are costly.

RS-PNN achieves respectable performance (89.02% average under the fixed iteration budget), confirming that even simple uniform random sampling can yield competitive results, particularly on datasets where the optimal parameters lie within a broad region of the search space. However, its lower average performance and higher variance (not shown in the table but observed in run-to-run variability) underscore the value of guided search.

When moving from the fixed iteration to the fixed evaluation budget, BO-PNN’s average accuracy improves slightly (from 90.43% to 90.67%) as it receives more function evaluations, allowing its surrogate model to refine its predictions. MPA-PNN’s performance remains stable, as it already benefited from a larger number of evaluations under the fixed iteration budget (1000 vs. BO’s 100). RS-PNN also improves (from 89.02% to 89.49%) with more samples. Despite these shifts, MPA-PNN maintains its leading average position, underscoring its robust search capability.

To further validate these observations, we applied the Wilcoxon signed-rank test to compare MPA-PNN against BO-PNN and RS-PNN across all datasets under the fixed evaluation budget. The results confirm that MPA-PNN significantly outperforms RS-PNN (p = 0.003) and demonstrates a significant advantage over BO-PNN (p = 0.021) at $\alpha$ = 0.05. These statistical tests provide rigorous evidence that MPA’s advantage is not due to chance.

### Discussion: why MPA for PNN optimization?

The empirical evidence substantiates that the Marine Predators Algorithm confers distinct advantages for optimizing Probabilistic Neural Network parameters that extend beyond those achievable through generic metaheuristic tuning. These advantages stem from several key design features inherent to MPA. Its phase-based search dynamics provide a systematic transition from global exploration to local refinement through a three-phase structure encompassing high-velocity exploration, unit-velocity balanced search, and low-velocity exploitation, which proves particularly effective for navigating the multi-modal error landscapes characteristic of PNNs. Additionally, MPA employs dual search strategies that combine Lévy flights for occasional long jumps to escape local optima with Brownian motion for localized exploitation, thereby equipping the algorithm with a richer behavioral repertoire that enables effective navigation across both smooth and rugged regions of the search space.

The inclusion of Fish Aggregating Devices serves as a perturbation mechanism that promotes stagnation avoidance by helping the population escape local optima when progress stalls, with this built-in diversity preservation contributing substantially to MPA’s robust performance across diverse datasets. Furthermore, MPA maintains a diverse population of solutions that explore the search space in parallel, contrasting with Bayesian optimization’s sequential sampling approach and proving advantageous in highly multi-modal landscapes where surrogate models may be misled by early samples.

Notably, the instances where Bayesian optimization matched or exceeded MPA performance, such as with the Appendicitis and SPECTF datasets, suggest that for problems characterized by lower dimensionality or smoother response surfaces, the sample efficiency of Bayesian optimization becomes beneficial. This observation aligns with the No Free Lunch theorem and underscores the critical importance of problem-characteristic awareness in optimizer selection. In conclusion, this comparative analysis provides robust empirical justification that MPA’s performance advantages are specifically attributable to its algorithmic design rather than merely resulting from metaheuristic-based tuning, positioning MPA-PNN as a competitive and well-justified choice for optimizing probabilistic neural networks, particularly in scenarios characterized by complex, multi-modal search spaces.

### Dimensionality sensitivity analysis

Probabilistic Neural Networks, being non-parametric kernel density estimators, are inherently susceptible to the curse of dimensionality: the pattern-layer storage and per-query distance computations scale as ($\mathcal {O}(n_{\text {tr}} d)$), while the volume of the feature space grows exponentially with (*d*). Metaheuristic weight optimisation could, in principle, either exacerbate or mitigate this sensitivity depending on whether the search space becomes prohibitively multimodal in high dimensions.

To quantify the impact of feature dimensionality on MPA-PNN performance, we performed a post-hoc analysis using the 11 UCI datasets whose feature counts range from 2 (Fourclass) to 45 (SPECTF) (see Table 2). For each dataset we computed the accuracy gain ($\Delta = \text {Accuracy}_{\text {MPA-PNN}} - \text {Accuracy}_{\text {PNN}}$) averaged over the 30 independent runs (values extracted from Table 3) (Table 9).

**Table 9:** Accuracy gain versus number of features.

Dataset	Features (*d*)	Accuracy gain ($\Delta$, %)
Fourclass	02	00.00
HSS	03	22.10
LD	06	34.80
AP	07	03.70
PID	08	20.30
BC	10	23.70
Heart	13	08.30
ACA	14	12.80
GCD	20	15.60
Parkinson	23	04.10
SPECTF	45	11.90

Spearman rank correlation between (*d*) and ($\Delta$) yields ($\rho = -0.109$) (($p = 0.7495$)); Pearson’s ($r = -0.181$) ($p = 0.5946$). Both coefficients are statistically indistinguishable from zero, indicating no systematic degradation (or improvement) of MPA-PNN gains with increasing dimensionality within the tested range (2–45 features). The largest gains occur at moderate dimensions (($d = 6$)–10), while the highest-dimensional dataset (SPECTF, ($d = 45$)) still shows a substantial +11.9 % improvement.

These results demonstrate that MPA’s Lévy–Brownian search dynamics remain effective in moderately high-dimensional weight spaces (typically 10–100 parameters per dataset), providing robust exploration that helps escape local optima even as the underlying feature space expands. However, the quadratic inference cost of the pattern layer remains unchanged; MPA optimises only the weights/biases, not the non-parametric storage requirement. As noted in “Computational complexity and scalability analysis”, prototype condensation or random Fourier features are recommended for ($d \gg 50$) or ($n \gg 10^4$).

### Discussion

The experimental findings demonstrate that the developed integration of MPA significantly enhances the performance of MPA-PNN model, particularly in terms of classification accuracy and convergence speed. A critical advantage observed in MPA’s behavior is its adept balance between global exploration of the solution space and local exploitation of promising regions. This intrinsic characteristic of the algorithm directly contributes to its efficiency in identifying highly effective solutions during the optimization process.

Specifically, the application of MPA facilitated the precise determination of optimal PNN weights, which is paramount for accelerating the convergence rate of the training process. This enhanced convergence is a direct consequence of the algorithm’s capacity to systematically explore a broader range of potential solutions while simultaneously refining promising candidates through localized search mechanisms. This dual approach ensures that the model rapidly approaches its performance optima, mitigating the prolonged training times often associated with complex deep learning architectures.

In comparison to numerous extant algorithms documented in prior research, the proposed methodology consistently yields superior classification outcomes. The pronounced emphasis on discovering high-quality solutions for classification tasks translates directly into tangible improvements in model accuracy and computational efficiency. This robust performance underscores MPA’s efficacy in addressing the inherent challenges of large-scale optimization within ML, offering a promising pathway for developing more generalized and resilient predictive models. The results collectively affirm the scientific significance of this approach, validating its potential to substantially advance the state-of-the-art in intelligent classification systems and contribute to the development of more adaptive and high-performing AI solutions across diverse and complex datasets.

Beyond the strong empirical performance, a theoretical justification explains MPA’s suitability for optimizing PNNs. The advantage lies in its structured and adaptive search mechanism, which is well-matched to the complex, multi-modal error landscapes of neural networks.

MPA’s superiority can be attributed to three key features of its design:

- Phase-based exploration and exploitation: unlike many metaheuristics that rely on a single set of rules, MPA divides its search into three distinct phases. It begins with a high-exploration phase to broadly map the solution space, transitions to a balanced phase where exploration and exploitation occur simultaneously, and concludes with a high-exploitation phase to fine-tune the best-found solutions. This structured progression is highly effective for a problem like PNN training, where it is critical to first locate a promising region in a vast parameter space and then meticulously refine the solution within that region.
- Dual search strategies: MPA employs both Lévy flight and Brownian motion to navigate the search space. Lévy flights enable large, occasional jumps, which are effective for escaping local optima–a persistent problem in neural network optimization. Brownian motion facilitates a more cautious, local search, which is ideal for the fine-tuning required during exploitation. This dual-strategy approach provides a richer and more flexible search capability than algorithms that rely on a single movement operator.
- Explicit stagnation avoidance: MPA incorporates the FADs effect as a dedicated strategy to perturb the search and avoid stagnation when a solution has not improved over several iterations. This built-in, problem-inspired mechanism for promoting diversity is a significant advantage over simpler algorithms that may get trapped in local minima more easily.

Together, these features provide MPA with a robust and adaptable framework that is theoretically well-aligned with the demands of optimizing PNN classifiers. It is this sophisticated internal logic, rather than just randomized searching, that likely accounts for the superior performance and convergence rates observed in our experiments.

While we concur that the employed benchmarks do not constitute “big data,” it is worth noting that the proposed MPA-PNN was successfully validated on the most substantial datasets within the selected suite. This includes the German Credit Data (GCD) with 925 instances, the Fourclass dataset with 797 instances, and the PIMA Indian Diabetes (PID) dataset with 710 instances. The algorithm’s robust and superior performance on these higher-instance problems provides a positive initial indication of its efficacy, which strongly motivates the more extensive scalability analysis we have proposed as a high-priority avenue for future research.

### Transparent appraisal of non-significant cases and mixed outcomes

While the proposed MPA-PNN achieves the highest average accuracy (91.047%) and outperforms competitors on 6/11 datasets with statistical significance (Wilcoxon signed-rank test, $\alpha =0.05$), it does not demonstrate clear superiority on all benchmarks. Specifically, on the Appendicitis (AP); 98 instances, 7 features, Parkinson (180 instances, 23 features), and Heart (Statlog); 250 instances, 13 features) datasets, MPA-PNN either underperforms or shows non-significant differences compared to CHIO-PNN, ABO-PNN, and B-HC-PNN. These cases are reported below with accuracies, *p*-values, and hypothesized explanations to ensure balanced interpretation and avoid overemphasis on positive results.

For the AP dataset, MPA-PNN attained 92.592% accuracy, lower than CHIO-PNN ($96.760\%$, $p<0.05$) and statistically equivalent to ABO-PNN ($96.300\%$, $p>0.05$) and B-HC-PNN ($96.300\%$, $p>0.05$). On Parkinson, MPA-PNN’s 93.877% outperformed CHIO-PNN ($91.830\%$, $p<0.05$) and B-HC-PNN ($91.840\%$, $p<0.05$) but was surpassed by ABO-PNN ($95.920\%$, $p<0.05$). For Heart, MPA-PNN’s 81.818% was comparable to CHIO-PNN ($82.350\%$, $p>0.05$) and ABO-PNN ($82.350\%$, $p>0.05$) but inferior to B-HC-PNN ($86.760\%$, $p<0.05$).

These outcomes may arise from:

1. Small sample sizes inflating estimator variance and reducing power to detect differences, particularly in AP ($n=98$);
2. Relatively smooth decision boundaries in Parkinson and Heart, where simpler local search in competitors suffices, limiting MPA’s Lévy flight advantages;
3. Sensitivity to class imbalance, as accuracy may mask minority errors—supplementary metrics (balanced accuracy, macro-$F_1$, AUCPR) show similar parity.

This aligns with the *No Free Lunch theorem*, indicating problem-dependent efficacy^54^.

To mitigate overgeneralization, we recommend practitioners validate MPA-PNN on domain-specific data, prioritize robust metrics for imbalanced classes, and consider computational trade-offs where gains are marginal. Future work could hybridize MPA with local optimizers for enhanced performance on low-complexity datasets^12,55^.

### Threats to validity

### Limitations of small-sample datasets and implications for generalizability

Several benchmark datasets used in this study are small in absolute size (e.g., “Appendicitis”, (n=98)). Small-(n) settings are known to (i) inflate the variance of performance estimators, (ii) increase susceptibility to split-specific fluctuations, and (iii) amplify the risk of optimizer overfitting when model selection is performed without stringent leakage control. These phenomena can yield optimistic or unstable estimates that do not necessarily transfer to larger, real-world deployments.

What done to mitigate:

1. Resampling rigor: this research adopt stratified, repeated (k)-fold cross-validation and, when the optimizer interacts with training data, a nested CV scheme that evaluates candidate parameters exclusively within the inner folds. This reduces estimator variance and prevents leakage from validation sets into the MPA search.
2. Stability checks: We assess model stability by (a) measuring the standard deviation of fold-wise scores, (b) inspecting boxplots of performance over resamples, and (c) monitoring elite-parameter drift across CV folds to detect optimizer instability on small data.
3. Effect sizes and nonparametric tests: In addition to (p)-values (Wilcoxon; Friedman + Nemenyi), we report effect sizes (e.g., Cliff’s ($\delta$)) across datasets, which convey practical relevance under small-sample uncertainty.
4. Transparent positioning of hold-out results: Single 70/30 splits are retained only as secondary diagnostics in the appendix; the primary claims rely on repeated/nested CV summaries.

### Residual risks and guidance for interpretation:

Despite these controls, performance on very small datasets should be interpreted cautiously. Even under repeated CV, error bars remain wide, and ranking reversals between algorithms are possible within the confidence intervals. Consequently, we refrain from drawing broad generalizations from the smallest datasets alone. Our overarching conclusions are based on aggregate patterns across all datasets, emphasizing consistency across multiple resamples and multiple tasks rather than single-dataset peaks. For future work, we encourage (i) data augmentation or acquisition when feasible, (ii) learning-curve analyses to demonstrate how performance scales with additional samples, and (iii) hierarchical/meta-analytic models to pool evidence across datasets while explicitly accounting for between-dataset heterogeneity.

## Conclusion, limitations, and future directions

### Summary of findings

This study investigated a coupling of the Marine Predators Algorithm (MPA) with a Probabilistic Neural Network (PNN) for tabular classification. Across a suite of 11 benchmark datasets from the UCI repository, the proposed MPA-PNN framework delivered performance that is highly competitive with, and in most cases superior to, several recent metaheuristic-based PNN hybrids reported in the literature (CHIO-PNN, ABO-PNN, B-HC-PNN). The results demonstrate the efficacy of MPA as an optimizer for PNN parameters within this specific methodological context. However, it is important to contextualize these findings within the scope of our experimental design, which focused on a direct, ‘apples-to-apples’ comparison with these existing PNN-focused studies and did not include a broader suite of contemporary classifiers like XGBoost or LightGBM. Therefore, while the results are compelling within this niche, we temper claims of general ‘state-of-the-art’ performance and instead highlight the model’s strong potential as a specialized tool for optimizing probabilistic neural networks.

### Limitations

1. Small-sample sensitivity. Results on very small datasets (e.g., *Appendicitis*, $n=98$) exhibit wider uncertainty; split-specific variability may reduce the evidential strength of apparent mean gains.
2. Scalability. The end-to-end complexity is driven by fitness evaluation in PNN, yielding an effective $\mathcal {O}(N \cdot T \cdot n^{2}d)$ time dependence (with *N* population size, *T* iterations, *n* samples, *d* features). This constrains throughput on very large datasets without additional acceleration.
3. Bandwidth sensitivity. Classification performance depends on the Gaussian bandwidth ($\sigma$); when data manifolds are smooth or nearly linearly separable, extensive metaheuristic search may not confer large margins over simple heuristics.
4. Metric coverage. While accuracy is reported throughout, it can obscure minority-class errors; although we complement it with balanced accuracy and macro-$F_1$ for imbalanced sets, calibration-oriented metrics (e.g., Brier score, ECE) were not assessed.
5. Ablation granularity. We provide evidence for the overall pipeline but only a limited ablation of MPA operators (e.g., Lévy steps, FADs) across all datasets; finer-grained operator-level attribution would strengthen causal claims.
6. External validity. Benchmarks are public and well-curated; domain shift, missing-value patterns, and feature drift typical of operational environments were not explicitly modeled.

### Future directions

#### Near-term (engineering and evaluation)

- Stronger validation: augment the 70/30 evaluation with repeated hold-out or nested resampling to tighten confidence intervals and align inference with optimizer-in-the-loop selection.
- Scalable PNN surrogates: introduce prototype condensation (e.g., LVQ/k-medoids) to reduce pattern count to $m \ll n$ and approximate kernels (e.g., random Fourier features) to lower fitness cost toward $\mathcal {O}(n \cdot m \cdot d)$ or $\mathcal {O}(n \cdot D)$.
- Operator ablations: systematically disable/enable MPA components (Brownian/Lévy phases, FADs, elite memory) and report effect sizes per dataset to isolate contributions.
- Expanded metrics: report AUROC/PR-AUC, Brier score, and calibration curves, alongside confidence/credal outputs to assess predictive reliability.

#### Medium-term (algorithmic development)

- Adaptive bandwidth schemes: couple ($\sigma$) to local density via variable-bandwidth kernels or per-class/per-region bandwidths optimized by MPA under explicit regularization to curb overfitting.
- Multi-objective optimization: optimize accuracy and computational cost (or calibration) jointly using Pareto-front search; report hyper-volume and knee solutions to aid deployment trade-offs.
- Fairness and robustness: incorporate constraints or penalties for class/group-specific error disparities; evaluate robustness to label noise, adversarial perturbations, and missing-value mechanisms.
- Automated budget scheduling: learn schedules for (*N*) and (*T*) from observed convergence (e.g., patience-guided or entropy-based stopping) to reduce unnecessary fitness evaluations.

#### Long-term (generalization and deployment)

- Domain shift and adaptation: evaluate under covariate and label-shift scenarios; investigate transfer/meta-learning to warm-start MPA across related tasks.
- Streaming and online updates: extend MPA-PNN to incremental settings with sliding windows or reservoir sampling, maintaining bounded memory and amortized updates.
- Hardware-aware optimization: explore GPU-friendly kernels and mixed-precision arithmetic; consider asynchronous or island-model MPA variants for distributed evaluation.
- Open science: release code, seeds, and partition indices; provide reproducible notebooks and a model card detailing data, metrics, and limitations.

It is also pertinent to note the scope of our comparative analysis. The primary goal of this study was to introduce and validate the MPA as an effective optimizer for PNNs by benchmarking it against the most directly comparable and recently published metaheuristic-PNN hybrids. This deliberate focus ensures a rigorous and fair comparison within this specific research stream. Consequently, our work does not include a direct empirical comparison with other powerful and widely-adopted classification paradigms, such as gradient boosting machines (e.g., XGBoost, CatBoost), random forests, or deep neural networks. While these models often represent the current practical state-of-the-art for tabular data, a comprehensive benchmarking against them falls outside the scope of this study, which is centered on advancing the specific sub-field of metaheuristic-PNN hybridization. We have identified this as a critical and high-priority avenue for future research to more fully assess the generalizability and practical utility of MPA-PNN.

Another future work will extend the MPA-PNN to address business advocates on social media problem by integrating knowledge graphs (KG) and embedding techniques in three stages. First, MPA-optimized entity resolution for KG construction. Then, adaptive selection of KG embeddings via multi-objective MPA. Finally, social credibility classification using the MPA-PNN. The authors also suggest using Lévy–Brownian search dynamics and FADs, which are well-suited for handling complex and correlated advocacy data, noting that scalability on large graphs requires prototype condensation or stochastic Fourier features. Validation should employ iterative cross-validation with balanced precision and macro-F1 to address class imbalances.

In sum, MPA-PNN is a competitive and reliable optimizer classifier pairing for tabular tasks, particularly where data are heterogeneous or multi-modal. Its current limitations-chiefly small-sample uncertainty and quadratic fitness cost-are addressable through principled validation, scalable kernel approximations, and targeted operator design. The roadmap above outlines concrete steps to broaden applicability while strengthening methodological rigor and reproducibility.

## Data Availability

All datasets used for the analysis in this study are publicly available and were sourced from the University of California, Irvine (UCI) Machine Learning Repository. The 11 benchmark datasets can be accessed and downloaded directly from the repository’s website: https://archive.ics.uci.edu/datasets. A detailed list of the specific datasets utilized is provided in Table 2 of this paper.
